# An evaluation of family-based treatment for restrictive-type eating disorders, delivered as standard care in a public mental health service

**DOI:** 10.1186/s40337-021-00498-2

**Published:** 2021-10-29

**Authors:** Mette Bentz, Signe Holm Pedersen, Ulla Moslet

**Affiliations:** Child and Adolescent Mental Health Center, Copenhagen University Hospital – Mental Health Services CPH, Bispebjerg Bakke 30, 2400 Copenhagen NV, Denmark

**Keywords:** Anorexia nervosa, Family-based treatment, Standard care, Outcome, Effectiveness

## Abstract

**Background:**

Family-based treatment (FBT) has demonstrated efficacy for anorexia nervosa (AN) in youth in randomized, controlled trials. It is important to assess if it shows a similar effectiveness when implemented in standard care.

**Aim:**

To evaluate outcomes of FBT for restrictive-type eating disorders, delivered as standard care in a public mental health service. Outcomes are remission, frequency of hospital admissions and day-patient treatment, and frequency of other adaptations within 12 months from commencement of treatment. Second, to compare the collaborative clinical decisions of successful treatment in standard care made by family therapist at the end of treatment, with more objective definitions of recovery.

**Methods:**

The design is a prospective, uncontrolled study of a consecutive series of patients with restrictive-type eating disorders, treated with FBT in a specialty unit at the Child and Adolescent Mental Health Centre in the Capital Region of Denmark.

**Results:**

FBT was successfully completed within 12 months by 57% of participants, and 47% completed with 20 sessions or fewer. Weight restoration was achieved by 75% within 12 months, and 46% achieved both normalisation of body weight and behavioural symptoms of AN within 12 months. A total of 20% needed intensified treatment. All aspects of remission were often not present simultaneously, and the collaborative clinical decisions of successful treatment only partly aligned with other parameters of remission.

**Conclusion:**

FBT showed good results when implemented as standard care, and it can be adapted to the specifics of local service organisation without compromising effectiveness.

## Plain English summary

We evaluated the outcome of family-based treatment for young people with anorexia nervosa and similar restrictive-type eating disorders, treated in a public mental health service with easy access to treatment. More than half of the young people had successfully terminated treatment within 12 month, and three quarters were weight-restored within 12 months although some were still in treatment. However, one in five young people needed a period of more intensive care. The study suggests that this form of treatment is effective outside of research trials and can be successfully implemented in diverse settings. While this treatment is effective for a majority, we need more knowledge about the needs of those who do not benefit enough, and we need information on how to identify them early in treatment.

## Introduction

Family-based treatment (FBT) focussing on anorexia nervosa (AN) has been established as an efficacious treatment for AN in children and adolescents [1, 2], although quantity and quality of the evidence still leaves something to be desired [3]. Recovery rates tend to fall between 30–40% across randomized studies at the end of treatment (EOT) and somewhat higher at follow-up [1, 2].

As FBT has contributed significantly to the treatment of AN, a disorder that has otherwise had a poor prognosis [4], it is recommended as first choice of treatment for AN and similar restrictive-type eating disorders in young people [5–8]. The core tenets of this approach were developed at the Maudsley Hospital, London [9, 10], and was later manualised as FBT [11]. The FBT manual has guided implementation in our Danish service context, in line with national guidelines [12].

FBT has its roots in systemic and behavioural therapeutic approaches; it is symptom oriented and organized in three phases. Phase one focuses on parent empowerment and supports parents to take charge of refeeding and interrupting of disturbed behaviours. Phase two focuses on gradually returning age-appropriate control of eating to the youth. Phase three focuses on relapse prevention, returning to normal family life, and continuing development of age appropriate autonomy [11].

An evidence-based therapy such as FBT, however, may perform differently in standard care compared to delivery in the research setting of a randomized controlled trial (RCT) for a variety of reasons [13–15]. Moreover, subtle cultural differences in family structure, values around parenting, and expectations towards health care may differ, even between western, industrial countries, and these factors are of specific interest when considering FBT, because the main mechanism for change is parental empowerment. For instance, lack of alternatives in a public service may reduce parental empowerment, as may a strong emphasis on child autonomy. On the other hand, easy and free access to FBT and economic compensation may imply earlier intervention and thus better chances of recovery. Therefore, an important part of implementation is studying feasibility and effectiveness of delivering FBT in standard care, at sites beyond those where evidence was derived. This is the starting point of the present study.

To date, two studies on FBT for young people with AN compared the effectiveness of delivery in a research trial versus standard care setting. They demonstrated that speed and degree of weight restoration in standard care compared well to RCT settings [16, 17]. Notably, one study took place in a government funded public health care setting that resembles ours, albeit on a different continent [17]. The government funding entails free-of-cost service, which may lower the threshold for entering treatment and improve early intervention. However, it may also limit the number of available alternative services to choose from and thus interfere with parental autonomy.

A secondary motivation of the present study is to evaluate correspondence between different aspects of recovery. In standard care, where dose of FBT is not fixed, the decision to terminate is often made as a collaborative clinical decision between therapist and family; the collaborative clinical decision of successful treatment reflects the appraisal that treatment goals are met, and that the young person and the family can manage potential residual problematic thoughts and feelings on their own. This may happen before or after more objective measures of remission are reached, and it is not clear how the collaborative clinical decision of successful treatment maps onto more objective markers or remission.

What constitutes recovery in anorexia nervosa is, in fact, debated and a consensus on a definition has not been reached [18]. Couturier and Lock [19] recommend a combination of weight recovery (95% of expected body weight based on population means) and a global score within normal range on the Eating Disorder Examination (EDE) [20] in adolescent AN studies. Focussing narrowly on weight restoration seems less suited for evaluating effectiveness of FBT; as parents take care of weight recovery in phase one, the young person may reach normal weight quite early in treatment, while still be unable to care for his/her own nutritional needs. Thus, when evaluating the effectiveness of FBT, a clinically meaningful conceptualization of remission needs to include a successful phase two, i.e., that the young person resumes age-appropriate autonomy regarding eating and symptom management.

To sum up, FBT is recommended for AN in Danish national clinical guidelines, based on evidence from RCTs. However, it is important to confirm whether it shows a similar effectiveness outside the original research settings. Further, it is important to confirm its effectiveness when implemented as standard care in a public mental health service, and in other countries than those in which most of the evidence is demonstrated. This is important for patient families and health policy makers, and it may add to our understanding of the essential and effective components of FBT.

## Aim

Our aim is to evaluate outcomes of FBT to AN, delivered as standard care in a public mental health service. We evaluate outcomes with respect to remission, frequency of hospital admissions and day-patient treatment, and frequency of other adaptations within 12 months from treatment start.

As a secondary aim, we compare the collaborative clinical decision of successful treatment in standard care, made by family and therapist at EOT, with more objective definitions of recovery: degree of weight restoration, normalization of eating responsibility, level of dietary restraint, and return of menstruation.

### Hypothesis

We hypothesise that no less than 40% of participants reach remission at or before 12 months of FBT, in accordance with published controlled studies.

## Method

This is a prospective, uncontrolled study of a consecutive series of patients with AN, treated with FBT as standard care in a specialty unit at the Child and Adolescent Mental Health Centre in the Capital Region of Denmark (CAMHS).

### Treatment

In the study period, the CAMHS unit delivered specialised outpatient treatment to all referred and diagnosed youth under the age of 18 within a geographically defined area, with no direct cost to the families. There was close collaboration with a psychiatric inpatient unit and a unit for day-patient treatment, both for eating disordered young people, within the same centre. A short stay at a paediatric unit for immediate somatic stabilisation was also possible. The unit had offered FBT as the first line of treatment for approximately 10 years and received consultation from one of the authors of the FBT manual (J. Lock), as well as from the Maudsley Hospital where the model was developed. Therapists were psychologists, child and adolescent psychiatrists and nurses trained in FBT via courses as well as practicing with a more experienced team member, and they received weekly supervision by supervisors experienced in FBT. Sessions were 60 min long.

Several major differences compared with published RCTs of FBT are worth noting. First, the threshold for entering FBT treatment was low; families did not need to consent to randomisation between two treatments, there was no cost, and waiting time was guaranteed to a maximum of 30 days in the Danish health care system.

Second, CAMHS offered FBT to patients with a higher degree of medical complications than recommended for outpatient FBT in the manual (lower body weight, growth arrest and pubertal delay, pulse rate < 50 beats/minute). This was possible because inpatient and outpatient units were part of the same centre, admission was managed by senior consultants from both units, and a swift intensification could take place if the patient became unstable or weight loss was not stopped quickly in outpatient treatment. These patients were followed closely with somatic monitoring until stable weight gain was established. This affected the therapeutic stance of FBT as well. There is a continuum between presenting ideas, suggesting, advising and instructing. In instances of acute underweight, and / or when parents are paralysed in a state of crisis, the stance of the therapist was rather directive and specific (e.g., “*serve 6 meals a day, including full milk (or similar), child need sick leave from school and physical rest*”). This is counter to the parent empowering stance of standard FBT, where parents are supported to make their own decisions based on their experience and the knowledge shared by the therapist. In our view, effective FBT needs to balance the need to support distressed parents and achieve early weight gain, which is predictive of a good prognosis at EOT [21–24] with the intention of empowering parents to find their own solutions regarding renourishment. Care is taken to change the therapist´s stance when the acute crisis is ameliorated, and to support the parents´ own decision-making processes later in therapy.

Third, while families are usually not offered meal plans, in line with the manual, they are offered consultations with a dietitian trained in FBT, who guides parents regarding energy dense meals to promote weight gain. Most families utilised 2 dietetic consultations*.*

Fourth, the unit had a routine of taking stock at 4 weeks, 3 months and every 3 months until termination, where the clinician and the family reviewed changes in e.g., symptoms as part of the session. This was guided by a structured status questionnaire (see the Mesures section). It was followed by a team meeting or clinical round, where progress and potential obstacles for progress were discussed. We believe that a systematic monitoring of progress is an important part of sound clinical practice, especially in AN, where duration may impact prognosis, and ineffective treatment may at worst be maintaining AN [25].

Fifth, session contents regularly shifted from a more direct focus on treating AN to dealing with parental dilemmas or between-home-management. This was done either with or without the patient and siblings present. These needs might be more prevalent in our cultural context; parents in Scandinavian countries are found to praise their children’s independence more than parents in a range of other countries [26]. This may explain why a substantial subgroup of Scandinavian parents finds the task of curbing eating disordered behaviours in their child difficult to reconcile with their parenting values. These parents need support to reframe their active role as carers in the context of behaviours endangering their child´s health. Additionally, it is common for children of divorced parents in Denmark to divide their time between their respective parent´s household, and this increases the need to facilitate the parents´ coordination between the two homes. Devoting time for parental dilemmas is supported by evidence that parent coaching can improve outcomes in initial non-responders [21], and that parent-focused treatment are an effective alternative to family-based treatment [27].

Lastly, most parents in Denmark received financial compensation from the government to stay home caring for an ill child, as both parents most often worked outside the home, and this external factor may have aided the task of renourishment in phase one and thus supported the effectiveness of FBT.

### Sample

Inclusion criteria were typical and atypical AN according to the ICD-10 criteria, which were still in effect in Denmark at the time of the study (ICD-10 diagnosis of F50,0 and F50,1, in the following denoted AN)[28]; (According to ICD-10, typical AN (F50.0) is characterized by significant weight loss, restrictive eating, disturbance of body image and dread of fatness, and secondary endocrine and metabolic changes, e.g., loss of menstruation. Atypical AN (F50.1) resembles typical AN but does not fulfill all features of typical AN.) Further inclusion criteria were starting treatment during a period of 16 months (August 2018- November 2019), and giving informed consent to the study. In our region, patients were referred to adult Mental Health Services at the age of 18, if further treatment was needed. Exclusion criteria were referral to adult services or moving out of the geographical catchment area before 12 months of FBT, as these reasons for termination do not add to the knowledge base on the effectiveness of FBT. However, patients dropping out during 12 months for other reasons, and patients needing referral to other forms of care for ED, etc. residential treatment, were included in the study sample, as they represent patients for whom FBT did not seem to be a good match.

A total of 167 gave consent (73% of the total number of patients with an ICD-10 diagnosis of F50.0 or F50.1 in the targeted period). Ten patients moved out of the geographical region or turned 18 before 12 months of FBT without recovery. Thus, the sample evaluating 12 months outcome of FBT comprised 157 participants (Table 1); age range 10–17 years, 75% lived with both parents, 8.3% were males, and 91.7% females; proportion of trans/cisgender was not registered. Diagnoses were typical AN (F50.0) in 97 (61.8%) of participants, and atypical AN (F50.1) in 60 (38.2%)*.* The reasons for not fulfilling a diagnosis of typical AN was estimated weight loss below 15% of expected body weight (*N* = *28,* 46.7% of participants with F50.1), not reporting psychological symptoms (e.g., disturbed body image) (N = 23, 38.3%), or still menstruating (N = 9, 15%). Following the criteria of ICD-11 [29], amenorrhea is not part of diagnostic criteria, and thus 106 (67.5% of all participants) would be classified as AN, and 51 (32.5%) would be classified as Other Specified Feeding and Eating disorders (OSFED) due to not meeting one of the remaining criteria for AN. In parallel, following the criteria of DSM-5 [30], where amenorrhea is also omitted from diagnostic criteria, 106 (67.5% of all participants) would be diagnosed with AN, whereas 28 (17.8%) with weight within normal weight range would be diagnosed with atypical AN form of OSFED, and the remaining 23 (14.6%) would be diagnosed with OSFED.

**Table 1:** Participant characteristics and dose of family-based therapy (FBT)

	Total, N = 157	Recovery group, N = 90 (57.3%)	Prolonged group, N = 55 (35.0%)	Dropout group, N = 12 (7.6%)	Difference between recovery and prolonged groups^a^
Age at start in years, mean (SD, range)	14.3 (1.6, 10.3–17.5)	14.4 (1.6, 10.3–17.5)	14.2 (1.6, 11–16.8)	14.8 (1.0, 13.0–16.5)	ns
Diagnosis of typical anorexia nervosa (F50.0)^b^, N (%)	97 (61.8)	52 (57.8)	40 (72.7)	5 (41.7)	ns
Diagnosis of atypical anorexia nervosa (F50.1)^b^, N (%)	60 (38.2)	38 (42.2)	15 (27.3)	7 (58.3)	ns
Males N (%) / females N (%)	13 (8.3)/144 (91.7)	10 (11.1)/80 (88.9)	≤ 3 males	0 / 12 (100%)	ns
Comorbid psychiatric diagnosis, N (%)	42 (26.8))	20 (22.2)	20 (36.4)	≤ 3	ns
Parents living together, N (%)	117 (74.5)	65 (72.2)	45 (81.8)	7 (58.3)	ns
Intake percent of EBW, mean (SD, range)	84.2 (9.5, 64.0–109.5)	85.4 (9.1, 68.1–109.5)	81.9 (9.3, 64.0–101.0)	85.3 (11.9, 68.9–108.8)	Recovery group > prolonged group, *p* = .027
Intake percent of IEBW, mean (SD, range)	83.4 (8.4, 64.0–106.8)	85.2 (8.7, 67.2–106.8)	80.2 (7.0, 64.0–97.3)	85.5 (7.9, 75.7–97.5)	Recovery group > prolonged group, *p* = .001
Intake weight ≦ 85% of IEBW, N (%)	91 (58.0)	42 (46.7)	42 (76.4)	7 (58.3)	Recovery group > prolonged group, *p* < .0001
Intake weight ≦ 70% of IEBW, N (%)	4 (2.5)	≤ 3	≤ 3	0	
Intake resting heart rate < 50 beat/minute at intake, N (valid %)	18 (12.2)	9 (10.0)	8 (14.5)	≤ 3	ns
Intake EDE-global score, mean (SD, range)	2.8 (1.3, 0.1–5.4)	2.8 (1.3, 0.4–5.0)	2.8 (1.3, 0.2–5.4)	2.8 (1.7, 0.1–4.5)	ns
Number of sessions of FBT first 12 months, mean (SD, range)	20 (9, 2–46)	15 (6, 2–30)	27 (8, 7–41)^c^	19 (10, 9–46)	
Completed FBT with 20 or fewer sessions, N (%)	76 (48.4)	76 (84.4)	n/a	n/a	
Duration of treatment (FBT, plus potential time spent in intensifications) months, mean (SD, range)	10 (5, 1–27)	7.4 (2.8, 0.5–12.8)	16.7 (3.8, 13–27)^d^	7.3 (3.7, 1.9–12.5)	
FBT phase 2 at 12 months, N (%)		n/a	21 (38.2)	n/a	
FBT phase 3 at 12 months, N (%)		n/a	23 (41.8)	n/a	

^a^Mann-Whitney U Test for continuous outcomes, Chi Square test for dichotomous outcomes

^b^According to the ICD-10 classification which were in use in Denmark at the time of the study

^c^Excluding ≦ 3 participants followed with non-FBT family support and weight monitoring in addition to intensified treatment

^d^Excluding 9 participants not terminated treatment by the date this manuscript was finalized, and ≦ 3 participants followed with non-FBT family support and weight monitoring in addition to intensified treatment

ns: not statistically significant (*p* > .05)

EBW: Expected weight for height and gender, according to Danish norms

IEBW: Individual, expected body weight for height and gender, based on growth chart for the individual child

EDE: Eating Disorder Examination

Binge/purge behaviours was reported by 40 (25%). The F50.1 participants had a lower EDE-global score (mean 2.4, SD 2.4) compared to the F50.0 participants (mean 3.1, SD 1.2). Comorbid psychiatric diagnosis was seen in 27%; most frequent comorbid diagnoses in descending order was anxiety disorders, depressive disorders and autism spectrum conditions.

### Estimations of remission

The collaborative clinical decision of successful treatment was made by the family and the therapist in the conversation towards the end of phase 2, and in phase 3. Specifically, taking stock every three months prompted the therapist and family to discuss what still needs to be addressed before termination of therapy.

The collaborative clinical decision of successful treatment reflects the appraisal that the young person is well, and that the family can manage potential residual symptoms without further treatment.

Individual, expected body weight for each child or adolescent (IEBW) was based on his/her childhood growth trajectory z-score on weight-for-length/height, based on Danish norms [31], as this is standard in Danish pediatric health care. A medical doctor evaluated the pre-morbid growth trajectory of each child. In the present study, IEBWs at EOT or 12 months are reported as the weight corresponding to the sex and height at EOT or 12 months, respectively, according to the individually expected growth trajectory set by the medical doctor. In addition, we report expected body weight according to the population median for height and sex (EBW) in order to facilitate comparison with published FBT-studies.

Further, we include two behavioural indicators of remission: age-appropriate responsibility for eating, and intention of dietary restraint. Age-appropriate responsibility for sufficient and regular eating is reported collaboratively by the therapist and the family via a 4-point Likert-scale (*Parents have full responsibility for eating / Patient have growing responsibility for eating in limited areas / Patient have co-responsibility or is practicing increasing responsibility with support from parents / patient have main responsibility for eating (corresponding to the normal for his/her age)).* In parallel, intention of dietary restraint is reported by the patient when taking stock with the family, based on the following questions with wordings borrowed from the EDE-child interview: Restraint over eating (Over the past four weeks, have you deliberately been trying to cut down on what you eat?), Desire to lose weight (over the past four weeks, have you wanted to lose weight?), and Maintained low weight (*(if low weight)* Have you been trying to make sure that you do not put on any weight?). Answers reported frequency on a 7-point likert scale. We defined mild dietary restraint as an intention of restrictive eating less than 50% of days, and no intention of weight loss nor intention to maintain underweight for the last four weeks.

Lastly, we report the mean of three EDE items representing psychological symptoms, scored on a 7-point Likert scale (where 0 represent the lowest and 6 the highest intensity or frequency as per EDE instructions [20]): Importance of shape, Importance of weight, and Feeling fat. This is a crude estimation of level of psychological symptoms, in lieu of a full EDE-interview at termination. A mean score of 2 or less on these three items will be operationalized as normalization of psychological symptoms.

### Measures

To confirm diagnosis and estimate level of acute risk, all patients undergo an Eating Disorder Examination [20] by a psychologist, a somatic evaluation by a medical doctor, and parents are interviewed on development and symptoms before initiating treatment.

A structured status questionnaire was filled by family and therapist in collaboration, when taking stock at 4 weeks from start, 3 months from start and every 3 months until the final stage of treatment, and at treatment termination. The status questionnaire reported e.g., body weight, height, menstruation, type of treatment, main diagnosis and comorbid diagnoses, degree of responsibility for eating, diagnostic questions of the EDE interview [20], level of psychosocial functioning (school, friends) and self-reported quality of life. Questions borrowed from the EDE had the original response options regarding frequency or intensity, and the generic questions had response options on similar Likert scales. Note that this use of EDE questions did not share the diagnostic and psychometric validity of the EDE. Rather, as they were administered in the family conversation, it was a way of securing that all involved were on the same page regarding progress and perceived symptoms. The questionnaire also included the clinician’s ratings of motivation, alliance, and main therapeutic challenges. For those participants that terminated treatment before or at 12 months from start, result of status questionnaire at termination is reported. For those that hadn´t terminated treatment within 12 months, results of status questionnaire at 12 months are reported.

### Statistical procedures

Data were managed using REDCap electronic data capture tools hosted at The Capital Region of Denmark [32, 33]. We used SPSS, ver. 25, for descriptive statistics [34]. Participants were grouped according to the main outcome of terminating treatment successfully within 12 months. Differences between the two groups of interest were analyzed with Mann Whitney U-test, due to violation of normality for continuous outcomes, and with Chi-square test and Cramers V test for dichotomous outcomes. These group comparisons are exploratory, and we made no correction of alpha level for multiple comparisons. In case of ≤ 3 individuals or ≤ 3% in a cell of any tables, these numbers are not analyzed, and are denoted as “ ≤ 3” to protect anonymity.

## Results

### Missing data

From intake assessment, data on global EDE was missing for 7 (4.5%) participants. Data on heart rate was missing for 10 (6.4%) participants. At EOT or 12 months, data on behavioural aspects of remission (intention of dietary restraint and level of age-appropriate responsibility for eating, presence of binging, purging and compulsory exercise) were missing for 50 or 51 participants (32%). Data on psychological aspects of remission were missing for 71 (45%) participants. These variables are reported and denoted as “valid %” (percentage of those with available data) in Table 2.

**Table 2:** Aspects of remission at end of treatment or 12 months, whichever comes first

	Total, N = 157	Recovery group, N = 90 (57.3%)	Prolonged group, N = 55 (35.0%)	Dropout group, N = 12 (7.6%)	Difference between recovery and prolonged groups*
Outcome percent of EBW, mean (SD, range)	99.0 (8.5, 73.4–122.3)	99.6 (9.1, 73.4–122.3)	98.7 (7.2, 80.4–115.0)	96.4 (8.8, 87.1–115.9)	ns
Outcome percent of IEBW, mean (SD, range)	97.7 (6.8, 72.9–113.4)	98.7 (6.7, 72.9–113.4)	96.1 (7.0, 79.6–110.7)	97.2 (5.7, 87.1–109.9)	ns
Number of participants with outcome weight ≥ 95% of EBW, N (%)	109 (69.4)	65 (72.2)	39 (70.9)	5 (41.7)	ns
Number of participants with outcome weight ≥ 95% of IEBW, N (%)	117 (74.5)	69 (76.7)	39 (70.9)	9 (75.0)	ns
For girls: return of menstruation, N (valid % of all girls / valid% of girls after menarche)	81 (56.3/73.6)	52 (65/75.4)	23 (44.2/74.2)	6 (50/60)	ns
Returned to age appropriate eating responsibility, N (valid %)	75 (70.8)	48 (78.7)	24 (61.5)	3 (50)	ns
Intention of dietary restraint, last 4 weeks, N (valid%)	16 (15)	3 (4.7)	11 (20)	≤ 3	Recovery Group < prolonged group. *p* < ,0001
Recovered as per ≥ 95% of IEBW + eating responsibility + mild to no dietary restraint (and for girls after menarche: also return of menses), N (valid %)	48 (46.2)	32 (52.5)	14 (37.8)	≤ 3	ns
Participants reporting any episodes of binging, last 4 weeks, N (valid %)	≤ 3	≤ 3	≤ 3	≤ 3	
Participants reporting any episodes of purging, last 4 weeks, N (valid %)	≤ 3	≤ 3	0	0	
Participants reporting any episodes of compulsory exercise, last 4 weeks, N (valid %)	9 (5.7)	≤ 3	5 (9.1)	≤ 3%	
Mean score of three psychological ED symptoms < 2**, last 4 weeks, N (valid %)	63 (73.3)	43 (84.3)	18 (58.1)	≤ 3%	Recovery Group > prolonged group, *p* = .008

*Mann–Whitney U Test for continuous outcomes, Chi Square test for dichotomous outcomes

**3 psychological symptoms are questions from the Eating Disorder Examination: Importance of shape, Importance of weight, and Feeling fat

ns: not statistically significant (*p* > .05)

(valid %): indicates that there is missing data on this variable, and the number denotes the percentage of those participants with available data on this variable

EBW: Expected weight for height and gender, according to Danish norm

IEBW: Individual, expected body weight for height and gender, based on growth chart for the individual child

### Results of main aim: Evaluation of 12-month outcome

The mean body weight of the total sample was 99% of EBW. However, the distribution implied that 69% of the participants had reached a normal weight when defined as ≥ 95% of their IEBW, whereas the remaining 31% had a lower weight (Table 2).

### Recovered group

Ninety participants (57%) reached the collaborative clinical decision of successful treatment within 12 months of FBT (termed recovered group in the following) according to the consensus rating between therapist and family. They received a mean of 15 FBT sessions in a mean duration of 7 months. Of these, 74 (84% of recovered group, 47% of full sample) completed treatment with 20 or fewer sessions. However, a subgroup of the remitted young persons experienced challenging psychiatric symptoms other than ED; 12 (8%) needed treatment for other psychiatric conditions after completing ED treatment.

### Dropout group

Dropout on initiative of the family was seen in seven (5%) cases, and additional five participants (3%) was transferred to long term residential or home-based care under the municipal administration, indicating complex cases where e.g., comorbidity or family background made FBT unsuitable; taken together, FBT seemed to not be a good treatment match or not a sufficient treatment for 12 participants (8%) (termed dropout group in the following). The dropout group received a mean of 19 sessions in a mean duration of 7 months, and 75% reached normal weight range before leaving treatment.

### Prolonged treatment group

The remaining 55 participants (35%) continued FBT treatment after 12 months (termed prolonged group in the following). 21 young persons in the prolonged group (38%) were in phase two of FBT at 12 months, and 23 (42%) were in phase 3 of FBT at 12 months, indicating that treatment was progressing, but they needed longer time to reach complete recovery. A total of 31 (56% of prolonged group, 20% of total sample) reached the collaborative clinical decision of successful treatment in a mean duration of 16 months of treatment (range 13–25 months).

### Differences between recovered group and prolonged group

The prolonged treatment group did not differ from the recovered group in proportion of atypical AN diagnosis, ratio of males/females, or proportion of parents living together. Frequency of comorbid psychiatric diagnoses was also not statistically significantly different between groups, although there was a trend towards more comorbidity with a weak association in the prolonged group (*p* = .065, Cramers V = .154).

At the start of treatment, median % of EBW and % of IEBW were significantly lower for those in the prolonged group, and significantly more young persons in the prolonged group had an initial body weight below 85% of IEBW. However, groups did not differ on level of psychological symptoms, as expressed by their EDE-global scores.

The proportion of participants who experienced return of menstruation or who had resumed age-appropriate independence in eating were not significantly different between groups. And the proportion of participants who had reached 95% or more of EBW and of IEBW did not differ significantly between groups. However, a significantly larger proportion of participants in the prolonged group still reported intention of dietary restraint, and they significantly more often reported elevated level of psychological symptoms as expressed by intensity ratings on three questions borrowed from the EDE: Importance of shape (Over the past four weeks has your shape been important in influencing how you feel about yourself as a person?), Importance of weight (Over the past four weeks has your weight been important in influencing how you feel about yourself as a person?), and Feeling fat (Over the past four weeks have you “felt fat”?).

### Frequency of modifications to FBT

During the first 4 weeks of treatment, 144 families (92%) received standard outpatient FBT, whereas 11 (7%) was transferred to more intensive forms of care within the first 4 weeks (Table 3).

**Table 3:** Overview of treatment adaptations and additions

	Total, N = 157	Recovery group, N = 90 (57.3%)	Prolonged group, N = 55 (35.0%)	Dropout group, N = 12 (7.6%)
Standard FBT only	121 (77.1)	76 (84.4)	34 (61.8)	11 (91.7)
Any intensification (day treatment and/or hospitalization), N (%)	31 (19.7)	8 (8.9)	20 (36.4)	3 (25%)
Hospitalization once, N (%)	8 (5.1)	5 (5.6)	3 (5.5)	0
Day treatment course once, N (%)	13 (8.3)	3 (3)	8 (14.5)	< 3
Use of both day treatment and hospitalization, or hospitalization more than once, N (%)	10 (6.4)	0	9 (16.4)	< 3
Extended weighing session with YP, N (%)	11 (7)	7 (7.8)	4 (7.3)	0
Individual therapy for YP, N (%)	20 (12.7)	< 3	8 (14.5)	3 (25)

YP: young person

During the full period till EOT or 12 months, 121 (77%) was treated with FBT as the only treatment modality. In 11 (7%) cases, the weighing session with the young person was extended in an agreed and systematic fashion, where up to 50% of session time was devoted to individual conversation with the young person; this mode was carried out for a mean of 36% of treatment duration for this subgroup. In 20 (13%) of cases, young persons had individual therapy, based on team discussion and feedback / wishes of the family. This mode was carried out for a mean of 35% of treatment duration for this subgroup. Individual therapy was mainly cognitive-behavioural, and targeted either remaining psychological symptoms after initial weight restoration or comorbid symptoms interfering with response to FBT. It was delivered by a team member, coordinated by the FBT therapist and included some parent-only sessions or family sessions to maintain the FBT framework of empowering parents to help their child beat ED.

### Frequency of day-patient treatment or inpatient treatment

More than half of the sample (52%) completed treatment within 12 months with outpatient treatment alone (recovered group minus 9% who had intensifications). A total of 31(20%) was transferred to a more intensive mode of treatment within 12 months from start, and 60% of these returned to outpatient FBT, either for a period (22%) or for the remainder of their treatment (48%). A majority of these were in the prolonged treatment group; 20 (36%) of the prolonged group had intensified treatment, and nine received both types of intensification (hospital admission and day treatment).

### Results of secondary aim: comparing aspects of recovery

For those in the recovered group, whose treatment was deemed to be successful by therapist and family, it varied whether they met all potential criteria for full recovery; 77% was weight restored defined as ≥ 95% of IEBW, 79% had fully resumed age-appropriate responsibility for eating, 95% no longer reported intention of dietary restraint, 84% reported low levels of psychological symptoms, and 75% of girls after menarche had resumed menstruation. When combining three criteria of remission: weight restoration, independent eating, and mild to no dietary restraint, and for girls after menarche a fourth criterion: return of menstruation, 52% of recovered group and 46% of full sample met all these criteria for recovery at EOT or 12 months, whichever came first. Surprisingly, even in the prolonged group, 38% of participants met these criteria.

## Discussion

We evaluated FBT for AN in standard care, in an open design with no fixed dose, and in a large sample. Our findings add to the knowledge base that FBT can be implemented effectively in a public mental health service, and at other sites than those where the treatment was developed, manualised and tested; standard FBT yielded good outcome for 52% of patients within 12 months, as measured by the collaborative clinical decision of successful treatment made by family and therapist at EOT.

Within 12 months of FBT, 69% of our sample were weight restored, defined as ≥ 95% of EBW, and if compared to the young person´s own growth trajectory (IEBW), 75% were weight restored. This is in line with findings from a study comparing FBT administered in a randomized research setting and in a specialty clinical care setting; 57.1% of participants were weight restored within 12 months [16]. Direct comparisons are not warranted since treatment contexts differ, but we hypothesise that easy access and short waiting time to FBT in our service context may contribute to a high effectiveness, since longer duration of AN is a moderator of outcome in several studies [35, 36].

### Adaptations vs core tenets of the model

Several differences between the FBT treatment offered in our study and that in published RCTs are worth discussing. One is the absence of a fixed dose of FBT. The RCTs administered between 10—20 sessions [10, 37–41], and the effect of working with no fixed limit in FBT is unknown. The a priori knowledge of when therapy will end may increase momentum and motivation for change. On the other hand, knowing that longer treatment is available when your child is seriously ill may support parents in crisis. Our findings suggest that the effectiveness of FBT has not suffered from the absence of a fixed dose, since 84% of our recovered group (47% of full sample) completed treatment successfully with 20 or fewer sessions. Of note, the finding that a proportion of young people in the prolonged group did well on several aspects of remission at 12 months suggests that they might have fared well even in a format with a fixed dose within 12 months.

Another potential difference between FBT delivery in our study and in published RCTs is the readiness to suggest, advise and even instruct parents regarding renourishment in the beginning of phase 1. We are not able to conclude whether the increased readiness to suggest, advise and instruct parents enhanced effectiveness in the present study, but given the outcomes we suggest that it did not hamper effectiveness.

### Hospitalisation

In our study, 20% had either a psychiatric hospital admission, and/or day-patient treatment, typically for 12 weeks. Types of intensive care and the criteria for entering them differ greatly between treatment studies due to e.g., health care organisation; for instance, in the landmark RCT by Lock and colleagues [37], admission was into a paediatric unit for immediate somatic stabilisation, and this was used by only 15% of participants [37]. In contrast, in our service, longer psychiatric admissions were available, and reasons for entering might be e.g., attenuated family crisis or non-response to FBT. However, our findings along with other FBT studies suggest that no matter how well FBT is administered, a subgroup of young people will need access to more intensive care at some point. This may inadvertently evoke feelings of powerlessness in parents and dependence on professionals, and thus counteract the parental empowerment that is central to FBT. In our experience, involving parents across treatment levels and careful collaboration and coordination around service transitions may counteract this side effect of hospitalisation.

### Definition of recovery

Definition of recovery vary in FBT studies; Body weight ≥ 95% of EBW, and psychological symptoms within 1 SD of EDE-global is recommended [19, 42]. Several studies focus solely on weight restoration [16, 17], as it predicts broader outcomes [43], and simplifies comparisons across studies. We argue that specifically for FBT, where parents temporarily manage food intake, we need to include return to age-appropriate eating autonomy in our understanding of recovery, as it seems to be a prerequisite for returning to school, eating with peers, at sports, and resuming normal social development. Nevertheless, even the ability to eat sufficiently in an independent fashion may return gradually, as is the case for most other aspects of remission [44]. Indeed, not all the young people in our study who successfully completed FBT within 12 months had fully resumed independent eating, and some were still practicing with support from parents. It may be a specific strength of family therapy in the context of AN that treatment can be terminated successfully before all aspects of recovery are reached, because parents understand the disorder and their child’s needs and continue to support and monitor progress after termination. This may explain why the outcomes of FBT seem to improve between EOT and follow-up [37].

### What if standard treatment is not enough?

In sum, FBT seems to be an especially efficient treatment when it comes to weight restoration. Even in prolonged and dropout groups, many of the young people were weight restored at 12 months or at dropout. However, no clear guidelines exist in cases where treatment goals are not met by session 20 of FBT. For 20% of participants, a higher dose of FBT (> 12 months) was enough to reach a good outcome. This is in line with the findings of Wallis et al., who showed that continued FBT improved outcomes for those who had not remitted after 20 sessions [45]. Our findings suggest an initially lower % of IEBW is an important factor in treatment duration, and that those who need to gain more weight tend to need longer duration of treatment.

For another albeit small subgroup, however, weight restoration and exposure to normal eating with familial support seems to be insufficient to obtain independence and wellbeing. The criticism levelled towards FBT for not attending sufficiently to psychological symptoms of AN, and to the psychological issues potentially underlying AN [46–48], may be warranted when it comes to this subgroup. More research is needed, however, on how to identify those at risk of not benefiting enough from standard FBT. Time is essential here, because families and youth become gradually more exhausted and lose hope, when FBT doesn´t work, and this may contribute to a vicious circle maintaining ED.

## Limitations

While it is a strength for day-to-day clinical decision making that all FBT therapists participate in data collection, is may also be a limitation for rigor and consistency and increase the risk of missing data. Missing data rates for behavioural and psychological aspects of remission at EOT are very high. This is mainly due to organizational issues; Clinicians were not prompted to remember the status questionnaire when ending treatment as they were at the regular time points during treatment, and they may have found it less clinically meaningful when the therapeutic work was ending. In addition, a subgroup of participants was reluctant to answer these questions, e.g., from being feed up with the demands of treatment. Unfortunately, the missing data on a proportion of the sample at EOT weakens the conclusions regarding correspondence between the collaborative clinical decision of successful treatment and more objective definitions of recovery. Lastly, our use of selected EDE questions at EOT does not share the diagnostic and psychometric validity of the full EDE interview. Further, using these questions in a family conversation when taking stock of treatment constitutes a rather different context than a research interview with a neutral researcher. For these reasons, a full EDE interview would be preferable to establish psychological recovery.

## Conclusion

We evaluated outcomes of FBT to restrictive-type eating disorder, delivered as standard care in a public mental health service, in an open study with a large sample. We found that 57% successfully completed treatment within 12 months, and 48% did so with 20 or less sessions. An additional 20% completed treatment successfully with a longer duration of treatment. Weight restoration was achieved by 75% within 12 months, and 46% achieved normalisation of body weight as well as behavioural symptoms of AN within 12 months. A total of 20% needed intensified treatment as inpatients or day-patients for a period. FBT seems to be well suited to adapt to circumstances in different cultural contexts, in standard care, and in a multidisciplinary context.

## Data Availability

The dataset analyzed during the current study are available in anonymized form from the corresponding author on reasonable request.

## Abbreviations

AN: Anorexia nervosa
CAMHS: Child and Adolescent Mental Health Center, Copenhagen University Hospital - Mental Health Services CPH
EBW: Expected body weight
EDE: Eating Disorder Examination
EOT: End of Treatment
FBT: Family-based treatment
IEBW: Individually expected body weight
ICD-10: International Classification of Diseases, 10th edition
RCT: Randomized, controlled trial
SD: Standard Deviation
